# Insights into inflammation and implications for the pathogenesis and long-term outcomes of endometrial cancer: genome-wide surveys and a clinical cohort study

**DOI:** 10.1186/s12885-024-12630-x

**Published:** 2024-07-17

**Authors:** Jing Wang, Zhichao Chen, Yaozhen Lai, Zebiao Ma, Luanhong Wang, Pier Luigi Fiori, Ciriaco Carru, Giampiero Capobianco, Li Zhou

**Affiliations:** 1 Department of Biomedical Sciences, University of Sassari, Sassari, Italy; 2 Department of Obstetrics and Gynecology, Second Affiliated Hospital of Shantou University Medical College, Shantou, China; 3 Department of Cardiology, Second Affiliated Hospital of Shantou University Medical College, Shantou, China; 4 Department of Gynecologic Oncology, Cancer Hospital of Shantou University Medical College, Shantou, China; 5 Gynecologic and Obstetric Clinic, Department of Medicine, Surgery and Pharmacy, University of Sassari, Sassari, Italy

**Keywords:** Endometrial cancer, Inflammation, Mendelian randomization analysis, Prognosis, Nomogram, LASSO regression

## Abstract

**Background:**

Despite evidence showing a connection between inflammation and endometrial cancer (EC) risk, the surveys on genetic correlation and cohort studies investigating the impact on long-term outcomes have yet to be refined. We aimed to address the impact of inflammation factors on the pathogenesis, progression and consequences of EC.

**Methods:**

For the genetic correlation analyses, a two-sample of Mendelian randomization (MR) study was applied to investigate inflammation-related single-nucleotide polymorphisms involved with endometrial cancer from GWAS databases. The observational retrospective study included consecutive patients diagnosed with EC (stage I to IV) with surgeries between January 2010 and October 2020 at the Cancer Hospital of Shantou University Medical College.

**Results:**

The 2-sample MR surveys indicated no causal relationship between inflammatory cytokines and endometrial cancer. 780 cases (median age, 55.0 years ) diagnosed with EC were included in the cohort and followed up for an average of 6.8 years. Increased inflammatory parameters at baseline were associated with a higher FIGO stage and invasive EC risk (odds ratios [OR] 1.01 to 4.20). Multivariate-cox regression suggested that multiple inflammatory indicators were significantly associated with overall survival (OS) and progression-free survival (PFS) (*P* < 0.05). Nomogram models based on inflammatory risk and clinical factors were developed for OS and PFS with C-index of 0.811 and 0.789, respectively. LASSO regression for the validation supported the predictive efficacy of inflammatory and clinical factors on the long-term outcomes of EC.

**Conclusions:**

Despite the fact that the genetic surveys did not show a detrimental impact of inflammatory cytokines on the endometrial cancer risk, our cohort study suggested that inflammatory level was associated with the progression and long-term outcomes of EC. This evidence may contribute to new strategies targeted at decreasing inflammation levels during EC therapy.

**Supplementary Information:**

The online version contains supplementary material available at 10.1186/s12885-024-12630-x.

## Background

Endometrial cancer, originating from the endometrium, is a prevalent gynecological malignant tumor with rising incidence globally [1]. The incidence of endometrial cancer has increased markedly in the past three decades, particularly in high-income nations. The whole lifetime risk of endometrial cancer for a woman is roughly 3%, with a high number of annual deaths [2, 3]. Endometrial cancer is categorized into four stages according to the criteria of the International Federation of Gynaecology and Obstetrics (FIGO), with patients in the early stages seeing a lower mortality risk [4].

Unlike most other malignancies in America, endometrial cancer is rising in both morbidity and related death [5]. In 2024, an estimated 67,880 cases are diagnosed with endometrial cancers in the USA, resulting in 13,250 deaths [6]. Despite the average age of diagnosis being 63 years, evidence from the epidemiology indicated a sustained rise in cases among young women who wished to preserve their ability to have children, yet little wish came true in the standard line of therapy for EC [7]. Meanwhile, for newly diagnosed cases with endometrial cancer, the economic burden was unaffordable. The mean per patient per month total cost during the pre-treatment period was US$17,210 and US$ 6,859 during the line of therapy [8]. However, women’s ability to access timely and evidence-based health services is negatively related to local socioeconomic index. Women from low-income regions are more likely to develop poorly differentiated, aggressive endometrial carcinoma due to inadequate local healthcare services [9]. Therefore, Low-cost, accessible biomarkers are needed for screening and prognosis.

Although the etiology of endometrial cancer is not fully clarified, however genetics, obesity, metabolism and reproductive factors are known as the major underlying causes [4]. Pathogenic variants in mismatch repair genes (*MLH1*, *MSH2*, *MSH6*, and *PMS2*), *BRCA1*, and *BRCA2* contribute to genetic susceptibility to endometrial cancer [10, 11]. The International Cancer Society suggested that the risk of endometrial cancer (RR = 7.1, 95% CI: 6.3–8.1) increases with excessive obesity [12]. Obesity-induced insulin resistance raises circulating insulin growth factor-1 (IGF-1) and reduces sex hormone binding globulin (SHBG). Excessive levels of estrogen stimulate endometrial proliferation and promote the development of cancer [13].

Growing evidence reported the link between inflammation and risk of EC [14, 15]. Chronic inflammation contributes to malignant tumor progression and therapeutic resistance via the inflammatory tumor microenvironment (TME). Associations have also been found between inflammation immune biomarkers, such as C-reactive protein (CRP), systemic immune-inflammation index (SII), Neutrophil to Lymphocyte Ratio (NLR), mean platelet volume (MPV) and cancer risk [16–18]. Neutrophils are differentiated phagocytes that evolved as an evolutionary adaptation responsible for inflammatory response in vivo [19]. Lymphocytes, produced in the bone marrow, are immune cells that mainly consist of B and T lymphocytes [20]. MPV, an indicator of platelet size, can provide insights into platelet functions and has recently been investigated in connection with inflammation and thrombosis [21]. CRP is a hepatocyte-derived protein that functions as a biomarker for both infection and inflammation [22]. Finally, it is likely that the association between inflammation immune markers and cancer is explained by confounders such as lifestyle patterns or subclinical conditions. Here, we tested the causal association by Mendelian randomization analysis. This approach investigates the causal mechanisms of exposure and outcome according to Mendel’s laws of inheritance, using genetic variations as instrumental variables. Compared to traditional epidemiologic research methods, MR accurately assesses causality in the presence of unavoidable or uncertain confounders [23]. Current epidemiological studies of endometrial cancer are primarily observational, with only a few articles analyzing EC with by genetic correlation effects [24]. Furthermore, no previous study has comprehensively examined the associations between circulating inflammatory parameters and EC, and our study specifically addresses this matter.

In the present investigation, we aimed to address the genetic relation between circulating inflammatory cytokines and endometrial cancer via Mendelian randomization. Particularly, we conducted an observational retrospective study to assess the association between inflammatory indicators and cancer progression prognosis among patients with EC.

## Methods

### Two-sample MR analysis

We used a two-sample MR analysis to investigate the causal relationships between 41 inflammatory cytokines (Supplementary Data S1) and endometrial cancer from the GWAS of Finns and 17 cohorts [25, 26]. The inverse variance weighted (IVW) test with random effects was applied for the primary analysis process to estimate the causal effect of inflammatory cytokines and endometrial cancer [27]. Four additional MR models were performed as supplementary methods [28–30]. The summary statistics GWAS data for inflammatory cytokines and endometrial cancer can be assessed from https://gwas.mrcieu.ac.uk/.

### Study population

Women presenting to the Cancer Hospital of Shantou University Medical College with initial surgical resection of endometrial carcinoma were included from January 1, 2010, through October 15, 2020, with a follow-up range of 0.25 to 13.10 years. Our retrospective, longitudinal cohort study provided data on age, BMI, medical history, follow-up duration, FIGO stage of endometrial cancer, surgical procedure, baseline inflammatory parameters and other potential confounders. Inclusion criteria were women diagnosed with endometrial cancer and hospitalized for hysterectomy with bilateral salpingo-oophorectomy. We excluded subjects with the following features: incomplete data regarding OS and PFS (*n* = 13); history of chronic inflammatory diseases, autoimmune diseases or hematological diseases (*n* = 7); lost follow-up (*n* = 29); history of acute inflammatory diseases or surgeries within one month (*n* = 6); cases with radiotherapy or chemotherapy before blood testing (*n* = 11). A total of 780 patients were included in our study.

### Ethics approval and consent to participate

We reported our study strictly following the Strengthening the Reporting of Observational Studies in Epidemiology (STROBE) reporting guideline [31]. Our study was approved by the Cancer Hospital of Shantou University Medical College Ethical Review Authority (Approval No. 2,024,010). Since the study was designed as a retrospective observational study and patient privacy was highly protected, informed consent was unnecessary.

### Exposure, outcome, and other variables

We collected all data from the electronic medical record management system. The blood testing was performed within one week before surgery at the Department of Clinical Biochemistry, the Cancer Hospital of Shantou University Medical College. The complete blood counts, including white blood counts (WBC), lymphocyte counts (LC), monocyte counts (MC), platelet counts (PLT), CRP, platelet distribution width (PDW), MPV and plateletcrit (PCT) were retrieved from the electronic medical records. The relevant inflammation indexes were calculated as follows: the NLR was derived from the absolute neutrophil and absolute lymphocyte counts; the platelet-to-lymphocyte ratio (PLR) was derived from platelet and lymphocyte counts; The SII multiplied by neutrophil and then divided by lymphocyte counts.

Overall survival (OS) and progression-free survival (PFS) were considered primary and secondary endpoint events, respectively. The endpoint events were recorded by physicians at the Department of Gynecologic Oncology. Participants without endpoint events were followed up until October 31, 2020. The follow-up routine was regular from hospital discharge to three months for the initial three years and reduced to once a year after that.

### Statistical analysis

For the outcomes of Mendelian randomization analysis, we applied the Bonferroni correction adjusted *P* < 0.001 (0.05/41) as a threshold to reduce the risk of Type I errors and identify statistically significant causality [32]. In the present analysis, logistic regression models were applied to obtain odds ratio (OR) with confidence interval (CI) with inflammation parameters as independent variables and FIGO stage, histologic invasion of EC as dependent variables in the basic model. In a multivariable model, we further adjusted for age (continuous), BMI (continuous), history of diabetes (yes/no), hypertension (yes/no), menopause status (yes/no) and age of menarche (continuous) at baseline. Next, the associations between inflammatory factors and overall survival and progression-free survival of endometrial cancer were examined using hazard ratio (HR) and 95% CI in the Cox proportional hazards regression model.

According to risk factors identified by the multi-variable Cox regression analysis, Kaplan-Meier curves were generated to estimate OS and PFS that occurred during follow-up. Two prognostic nomogram models were developed using cumulative inflammatory risk and additional clinicopathological parameters to forecast 5-year and 10-year overall survival and progression-free survival [33]. We used a calibration curve and concordance index (C-index) to estimate the discriminative capacity of the nomogram model. We conducted the LASSO regression analysis to validate the association between inflammatory parameters and EC [34]. All variables in the previous analysis served as potential confounders, specifically those inflammatory parameters, were included in the model. We conducted all statistical analysis using R statistical software version 4.2.1 and GraphPad Prism for visualization. *P* < 0.05 for two tails were regarded as significant.

## Results

### Mendelian randomisation study

**Fig. 1:**
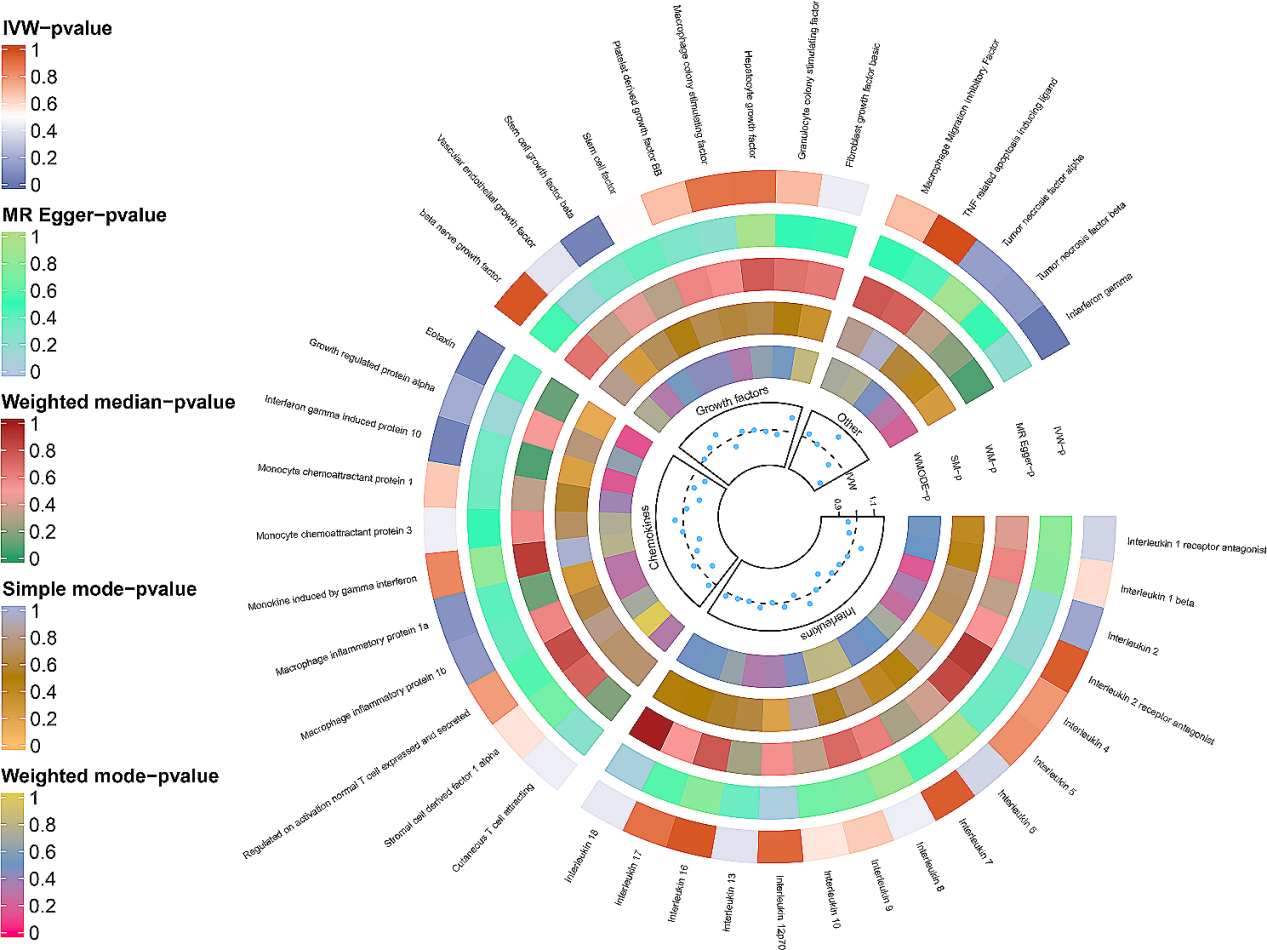
Circos plot of Mendelian randomization estimates for the association between inflammation-related SNPs and endometrial cancer

**Fig. 2:**
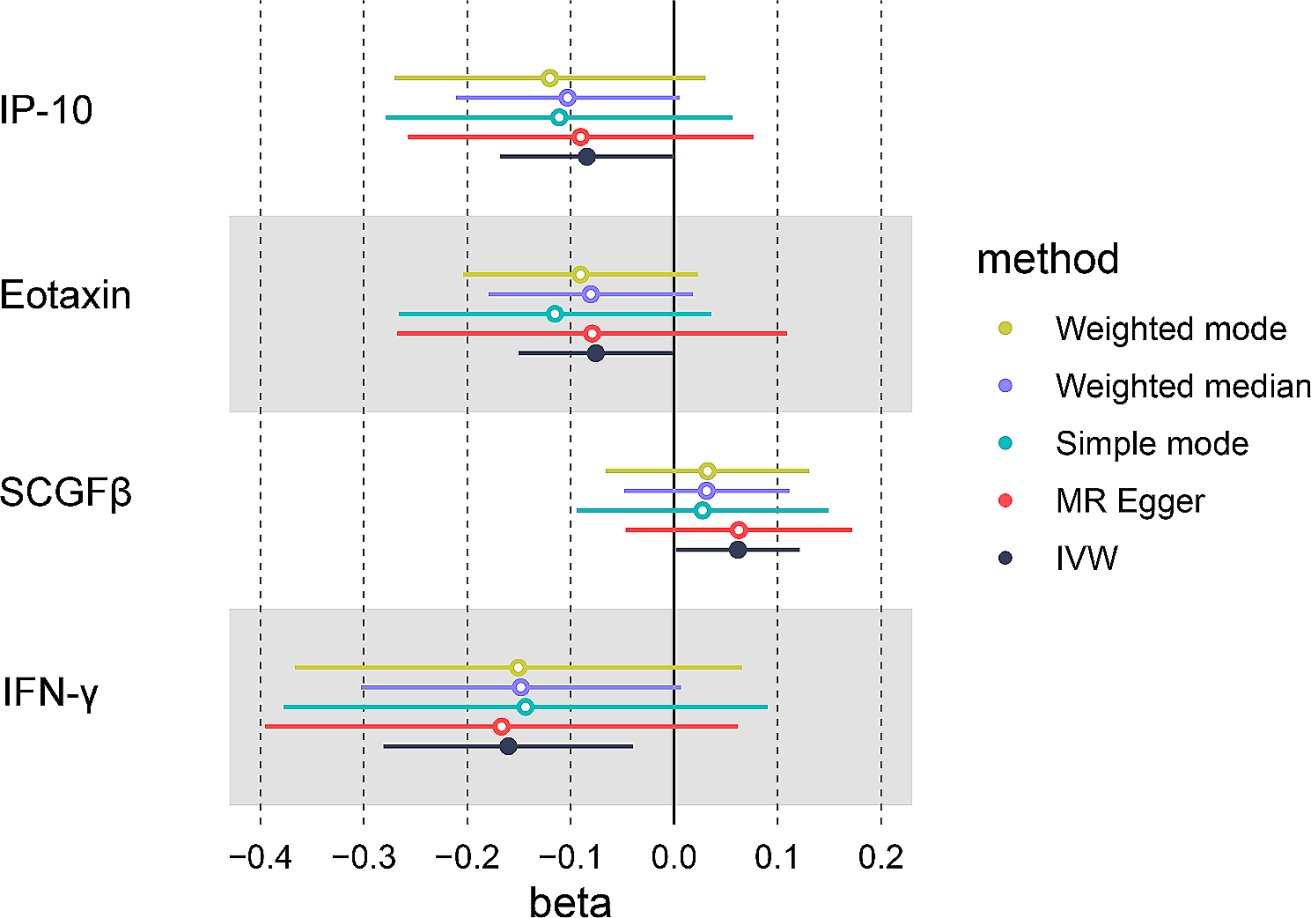
Forest plot of MR analysis for the causal effect of IP-10, Eotaxin, SCGFβ, and IFN-γ on endometrial cancer

A total of 365 SNPs with F statistics ranging from 11.16 to 789.15 were included in the analysis (Supplementary Data S2). 2- sample MR analysis indicated suggestive causal relationships of inflammatory cytokines, such as IL-10 (OR = 0.919; *P* = 0.049), Eotaxin (OR = 0.927; *P* = 0.047), IFN-γ (OR = 0.852; *P* = 0.009) and SCGF-β (OR = 1.064; *P* = 0.044) on endometrial cancer risk (Fig. 1). The results were similar in sensitivity analyses (MR-Egger regression, Cochran’s *Q*, and MR-PRESSO test) since either horizontal pleiotropy or heterogeneity were not detected (Supplementary Table S1). However, none of the inflammatory cytokines was causally related to endometrial cancer after Bonferroni correction. Supplementary Data S3 and Fig. 2 present the causal relationships between 41 inflammatory cytokines and endometrial cancer.

### Cohort study

#### Patient characteristics

The baseline characteristics of patients enrolled in the present study are shown in Table 1. 780 participants were diagnosed with endometrial cancer at a median age of 55.0 (range 25.0–79.0) years old. The average follow-up was 6.8 (range 0.3–13.1) years. The entire cohort consisted of an overweight and obese population (BMI ≥ 25.0 kg/m^2^, *n* = 381, 48.8%) and menopause (61.0% vs. 39.0%). More than 80% of women were diagnosed with FIGO Stage I/II endometrial cancer. For patients with postoperative chemotherapy or radiotherapy, the percentage was 27.8% and 21.8%. A large proportion of women had comorbid hypertension (336, 43.1%) and type 2 diabetes mellitus (149, 19.1%), respectively. Among them, 111 had endpoint events; 86 cases died during follow-up, and 44 cases experienced tumor recurrence.

**Table 1:** Baseline characteristics

Variable	Median (Range)	*N* (%)
**All**		780 (100)
**Age at diagnosis (years)** ^**1**^	55.0 (25.0–79.0)	
**BMI** ^**1**^	24.9 (15.6–49.0)	
**Age at menarche (years)** ^**1**^	16.0 (11.0–25.0)	
**Menopause**		476 (61.0)
**Hypertension**		336 (43.1)
**Diabetes**		149 (19.1)
**FIGO stage**		
I-II		634 (81.3)
III-IV		146 (18.7)
**Histologic invasion**		177 (22.7)
**Lymph node metastasis**		53 (6.8)
**Postoperative chemotherapy**		217 (27.8)
**Postoperative radiotherapy**		170 (21.8)
**Surgical procedure**		
Laparotomy		434 (55.6)
Laparoscopy		346 (44.5)
**Recurrence**		44 (5.6)
**Death**		86 (11.0)

#### Inflammation and the progression of endometrial cancer

**Fig. 3:**
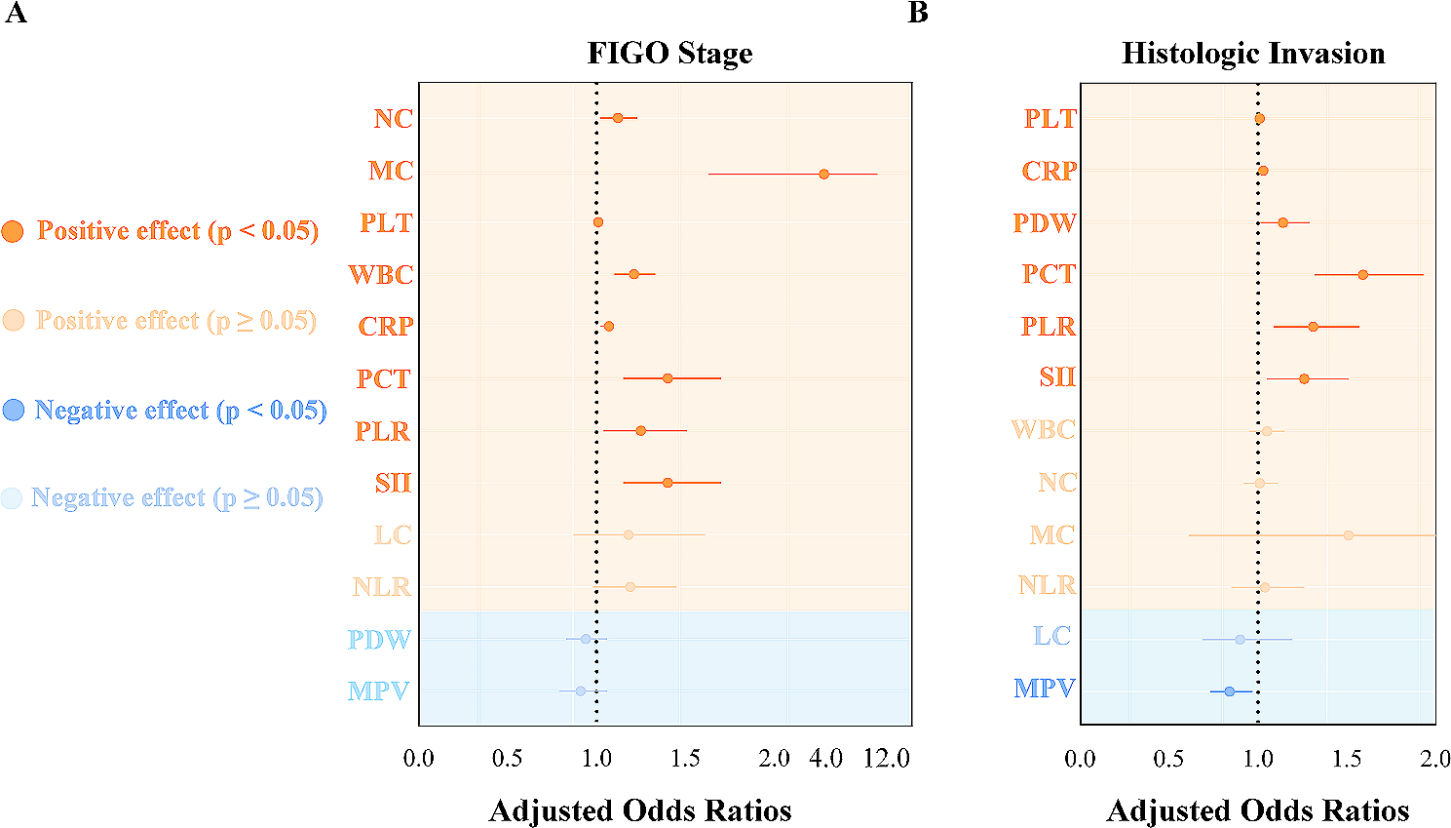
Multivariate logistic regression of inflammatory parameters and endometrial cancer risk

Table 2 shows the results of associations between inflammatory parameters and EC progression. In the unadjusted model, seven and six inflammatory indicators were associated with an increased risk of high-grade cancer stage (FIGO III/IV) and histologic invasion, while mean platelet volume was negatively related to histologic invasion risk in patients with EC. In a multivariate logistic regression model, the association remained undiminished and persistent after adjustment for potential confounders. The ORs of NC, MC, WBC, CRP, PCT, PLR and SII for high-grade cancer stage were 1.12 (95%CI, 1.02–1.23), 4.20 (95%CI, 1.66–10.65), 1.21 (95%CI, 1.10–1.33), 1.07 (95%CI, 1.02–1.11), 1.40 (95%CI, 1.15–1.70), 1.25 (95%CI, 1.04–1.51) and 1.40 (95%CI, 1.15–1.70), sequentially (*P* < 0.05) (Fig. 3A). PDW (OR 1.14, 95%CI, 1.01–1.29), MPV (OR 0.84, 95%CI, 0.73–0.97), PCT (OR 1.59, 95%CI, 1.32–1.93), PLR (OR 1.31, 95%CI, 1.09–1.57) and SII (OR 1.26, 95%CI, 1.05–1.51) were significantly associated with histologic invasion (*P* < 0.05) (Fig. 3B) (Supplementary Data S4).

**Table 2:** ORs for the association of inflammation indicators with tumor stage

	Model 1				Model 2			
	FIGO stage		Histologic invasion		FIGO stage		Histologic invasion	
	OR(95%CI)	*P*	OR(95%CI)	*P*	OR(95%CI)	*P*	OR(95%CI)	*P*
**NC**	1.05 (0.98, 1.12)	0.184	1.00 (0.93, 1.07)	0.887	1.12 (1.02, 1.23)	0.017	1.01 (0.92, 1.11)	0.839
**LC**	1.01 (0.77, 1.33)	0.930	0.98 (0.76, 1.27)	0.892	1.18 (0.87, 1.61)	0.283	0.90 (0.68, 1.19)	0.465
**MC**	2.36 (1.10, 5.06)	0.027	1.17 (0.55, 2.29)	0.693	4.20 (1.66, 10.65)	0.003	1.51 (0.61, 3.73)	0.369
**PLT**	1.01 (1.00, 1.01)	< 0.001	1.01 (1.00, 1.01)	< 0.001	1.01 (1.00, 1.01)	< 0.001	1.01 (1.00, 1.01)	< 0.001
**WBC**	1.15 (1.06, 1.26)	0.001	1.05 (0.97, 1.14)	0.214	1.21 (1.10, 1.33)	< 0.001	1.05 (0.95, 1.15)	0.321
**CRP**	1.07 (1.03, 1.11)	< 0.001	1.02 (1.00, 1.01)	0.035	1.07 (1.02, 1.11)	0.002	1.03 (1.01, 1.06)	0.020
**PDW**	0.99 (0.88, 1.10)	0.816	1.13 (1.01, 1.26)	0.043	0.94 (0.83, 1.06)	0.301	1.14 (1.01, 1.29)	0.046
**MPV**	0.93 (0.81, 1.06)	0.275	0.87 (0.76, 0.99)	0.028	0.91 (0.79, 1.06)	0.223	0.84 (0.73, 0.97)	0.014
**PCT** ^**a**^	1.36 (1.15, 1.61)	< 0.001	1.34 (1.14, 1.57)	< 0.001	1.40 (1.15, 1.70)	0.001	1.59 (1.32, 1.93)	< 0.001
**PLR** ^**a**^	1.29 (1.10, 1.51)	0.002	1.18 (1.01, 1.38)	0.019	1.25 (1.04, 1.51)	0.016	1.31 (1.09, 1.57)	0.004
**NLR** ^**a**^	1.13 (0.96, 1.33)	0.136	0.99 (0.84, 1.17)	0.917	1.19 (0.98, 1.45)	0.072	1.04 (0.85, 1.26)	0.739
**SII** ^**a**^	1.30 (1.10, 1.53)	0.001	1.14 (0.98, 1.33)	0.010	1.40 (1.15, 1.70)	0.001	1.26 (1.05, 1.51)	0.013

Model 1 was unadjusted. Model 2 was adjusted for age (continuous), age at menarche (continuous), BMI (continuous), menopause (yes/no), hypertension (yes/no), diabetes (yes/no)

Abbreviations: *NC*, neutrophil count; *LC*, lymphocyte count; *MC*, monocyte count; *PLT*, platelet count; *BMI*, body mass index; *WBC*, white blood cell count; *CRP*, C-reactive protein; *PDW*, platelet distribution width; *MPV*, mean platelet volume; *PCT*, plateletcrit; *PLR*, platelet-lymphocyte ratio; *NLR*, neutrophil-lymphocyte ratio; *SII*, systemic immune-inflammation index

^a^ORs for each sd increment

#### Inflammation and the prognosis of Endometrial Cancer

**Fig. 4:**
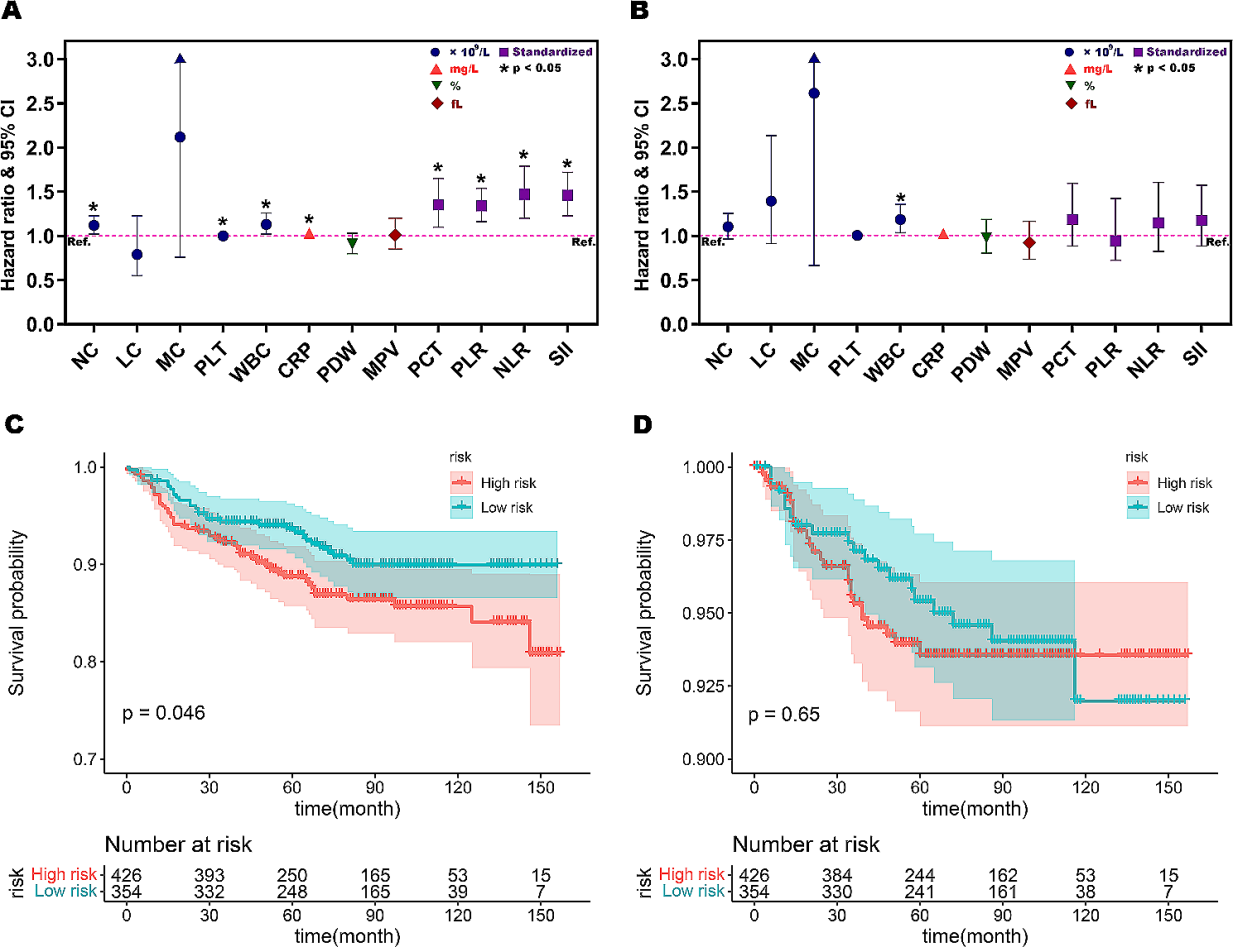
Hazard ratios of inflammatory factors for OS (**A**) and PFS (**B**), Kaplan–Meier curves of OS (**C**) and PFS (**D**) according to inflammation risk

Univariate cox-regression analyses demonstrated that seven and one inflammatory indicators were involved with overall survival and progress-free survival (*P* for trend < 0.05) (Supplementary Table S2 and Table S3). In multivariate analyses, WBC (HR 1.13, 95%CI 1.02–1.26), PCT (HR 1.35, 95%CI 1.10–1.65), PLR (HR 1.34, 95%CI 1.16–1.54), NLR (HR 1.47, 95%CI 1.20–1.79) and SII (HR 1.46, 95%CI 1.23–1.72) were independent predictors of OS (*P* < 0.05) (Table 3; Fig. 4A).Conversely, WBC (HR 1.18, 95%CI 1.03–1.35) was the sole independent risk factor for PFS (*P* < 0.05) (Table 3; Fig. 4B).

**Table 3:** Multivariate-cox regression of endpoint events (*n* = 780)

Variable	Endpoint Events									
	OS					PFS				
	Coef	HR	95% CI	*P*	Risk	Coef	HR	95% CI	*P*	Risk
**NC**	0.12	1.12	1.02, 1.23	0.016	high	0.09	1.10	0.96, 1.25	0.170	high
**LC**	−0.24	0.79	0.55, 1.23	0.189	low	0.33	1.39	0.91, 2.13	0.131	high
**MC**	0.73	2.12	0.76, 5.92	0.150	high	0.96	2.61	0.66, 10.30	0.171	high
**PLT**	0.00	1.00	1.00, 1.00	0.001	neutral	0.00	1.00	1.00, 1.01	0.144	neutral
**WBC**	0.13	1.13	1.02, 1.26	0.021	high	0.16	1.18	1.03, 1.35	0.019	high
**CRP**	0.03	1.03	1.01, 1.05	< 0.001	high	0.02	1.02	0.99, 1.05	0.281	high
**PDW**	−0.10	0.91	0.80, 1.03	0.128	low	−0.03	0.97	0.80, 1.18	0.756	low
**MPV**	0.10	1.00	0.85, 1.20	0.910	neutral	−0.08	0.92	0.73, 1.16	0.486	low
**PCT**	0.30	1.35	1.10, 1.65	0.005	high	0.17	1.18	0.88, 1.59	0.266	high
**PLR**	0.29	1.34	1.16, 1.54	< 0.001	high	0.01	0.94	0.72, 1.42	0.941	low
**NLR**	0.38	1.47	1.20, 1.79	< 0.001	high	0.13	1.14	0.82, 1.60	0.437	high
**SII**	0.38	1.46	1.23, 1.72	< 0.001	high	0.16	1.17	0.88, 1.57	0.283	high

Multivariate cox-regression model was adjusted for age (continuous), age at menarche (continuous), BMI (continuous), menopause (yes/no), hypertension (yes/no), diabetes (yes/no)

We conducted the OS and PFS curves using Kaplan-Meier survival analyses and the log-rank test. An inflammatory risk score was calculated based on the risk for the endpoint events. Patients with EC were categorized into low-risk and high-risk groups based on the median risk score. Survival curves showed a better overall survival rate in the low-risk group than in the high-risk group, suggesting the prognostic value of the inflammation risk score in OS (*P* = 0.046) (Fig. 4C). However, survival analysis for PFS indicated that there was no significant difference in the two risk group (*P* = 0.65) (Fig. 4D).

#### Establishment of prognostic model in patients with endometrial cancer

**Fig. 5:**
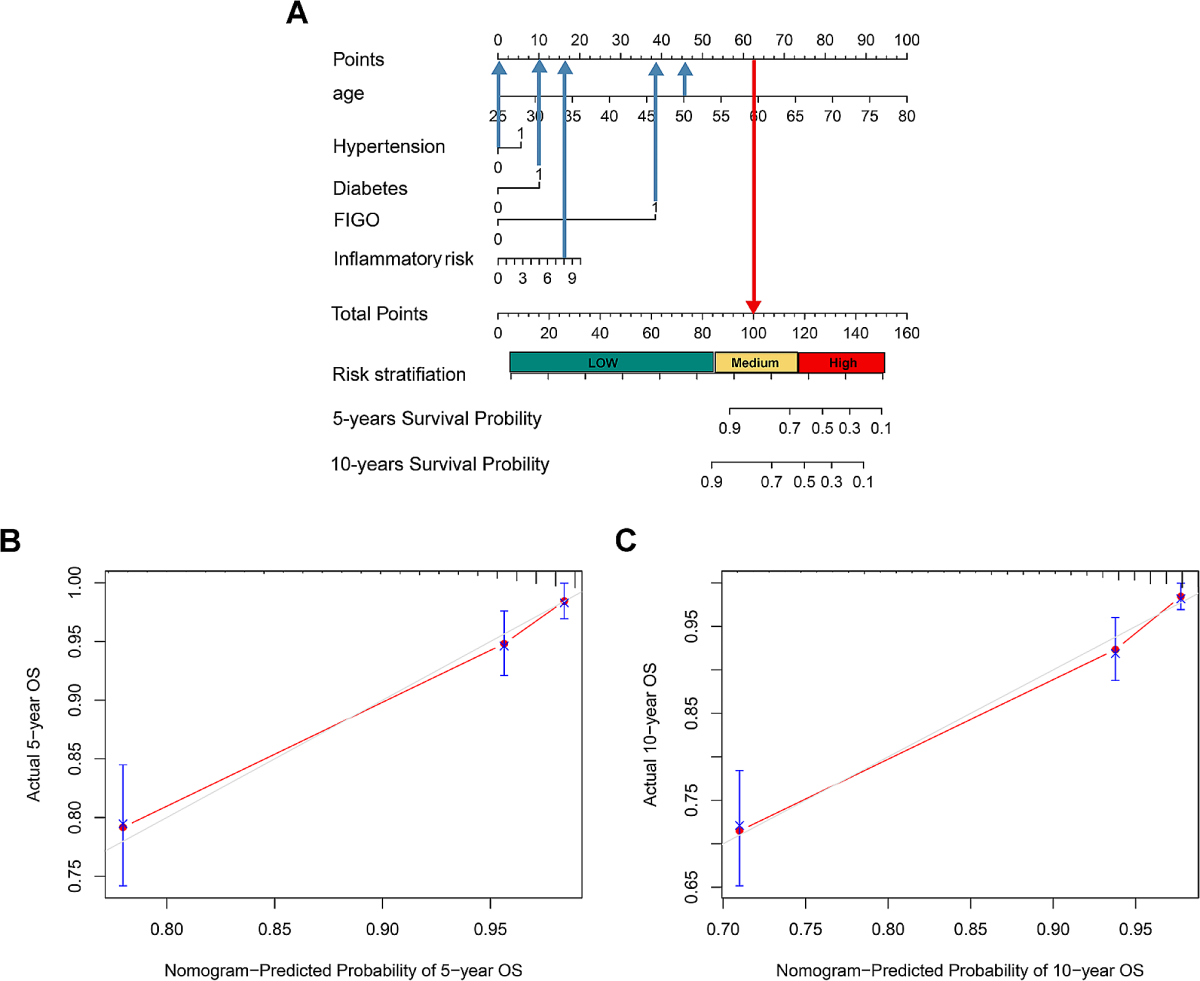
Nomogram for estimating 5 or 10 years OS probility for cases with endometrial cancer (**A**), Calibration curves of the nomograms of 5 (**B**) or 10 year (**C**) OS prediction

A nomogram model included four clinical prognostic factors, and an inflammatory risk score was developed to predict the likelihood of 5-year and 10-year endpoint events (Fig. 5A). In nomogram models, cases with a history of hypertension or diabetes were classified as a value of “1”, or otherwise were assigned a value of “0”. The FIGO stage was also categorized into “0” and “1” (0 = I/ II, 1 = III/IV). The concordance index (C-index) of the nomogram for OS prediction was 0.81. The calibration curve confirmed good consistency of the predicted OS with the actual OS (Fig. 5B, C). The C-index of the nomogram model for PFS was 0.79. Supplementary Table S4 shows the nomogram model for PFS and the calibration curves.

#### Validation of the impact of inflammatory parameters on endpoint events

**Fig. 6:**
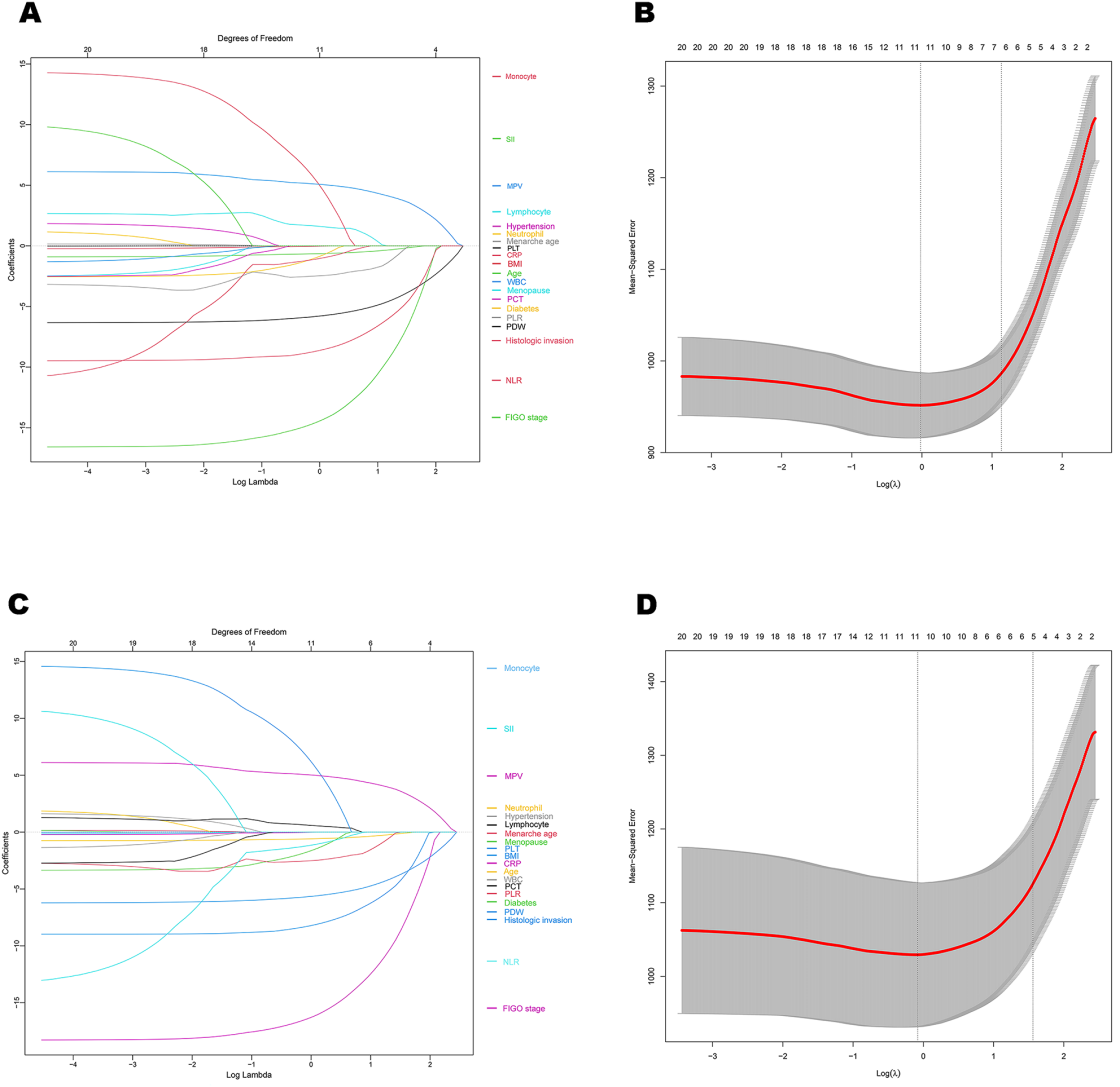
LASSO coefficients of inflammatory and clinical factors according to OS (**A**) and PFS (**C**), selection of the influencing variables by LASSO regression in the outcomes of OS (**B**) and PFS (**D**)

LASSO regression was performed to validate the impact of clinical factors and inflammation parameters on OS and PFS. As shown in Fig. 6, the optimal lambda were 0.895 for the OS model and 0.873 for the PFS model, respectively. 11 inflammatory parameters with predictive prognostic value were selected, and their regression coefficients were presented in Table 4.

**Table 4:** Lasso regression of endpoint events (*n* = 780)

OS		PFS	
Factors	LASSO ceofficient	Factors	LASSO ceofficient
**Age**	−0.66	**Age**	-0.60
**Diabetes**	−1.05	**Diabetes**	-1.86
**FIGO stage**	−14.65	**FIGO stage**	-16.55
**Lymphocyte**	1.64	**Histologic invasion**	-8.35
**Monocyte**	5.69	**Lymphocyte**	0.65
**PLR**	−2.50	**Monocyte**	7.04
**NLR**	−1.13	**PLR**	-2.55
**CRP**	−0.04	**NLR**	-1.26
**PDW**	−5.84	**CRP**	-0.02
**MPV**	5.11	**PDW**	-5.71
**Histologic invasion**	−0.87	**MPV**	5.07

## Discussion

### Main findings

Our study utilized an observational retrospective study design and a two-sample Mendelian randomization analysis to address the impact of inflammation on the pathogenesis, progression and consequences of endometrial cancer. The MR results suggested associations between the inflammatory cytolines IL-10 (OR = 0.919; *P* = 0.049), Eotaxin (OR = 0.927; *P* = 0.047), IFN-γ (OR = 0.852; *P* = 0.009), and SCGF-β (OR = 1.064; *P* = 0.044) with EC. However, these associations were not statistically significant after Bonferroni correction (to reduce false-positive causality) *P* < 0.001 (0.05/41). In the cohort study, preoperative circulating inflammatory parameters reflected the outward manifestation of the immune response to cancer progression and invasion. Furthermore, these preoperative indicators of inflammation have shown predictive value for the long-term postoperative outcomes of patients with EC. Our study provides novel evidence regarding inflammatory factors and genesis, progression and prognosis of endometrial cancer.

### Comparisons with previous studies

A previous MR investigation [35] reported a negative association between Interleukin-1 Receptor Antagonist (IL-1Ra) and endometrial cancer risk (OR = 0.86, *P* = 2.23 × 10^− 4^). In contrast, our results indicated no significant causal association between them. Different populations of inflammatory cytokines and multiple correction methods probably contributed to inconsistent findings. Furthermore, observational studies indicated that the association was controversial [36, 37]. Consequently, the results of MR analyses could be spurious causality due to factors such as population and genomic pleiotropy. More diverse population samples are warranted for genomic analysis to ensure the stability and generalisability of the evidence.

The findings of our clinical survey are consistent with prior observational cohort studies [38–41], suggesting the association between inflammation and endometrial cancer. Nevertheless, we applied Mendelian randomization analysis to assess the link between inflammation and endometrial cancer risk, which eliminated the bias of confounding factors such as environmental factors and lifestyle behaviors on the results. Moreover, we examined the association of all common circulating inflammatory parameters with EC, in contrast to prior studies focusing on a single indicator. It helps to draw comprehensive and objective conclusions.

### Mechanisms

Approximately 20% of human malignancies are linked to persistent inflammation resulting from infections, exposure to stimulus, or autoimmune illnesses [42]. For instance, chronic inflammation could contribute to the development of cancers, including gastric lymphoma from Helicobacter pylori infection, colorectal cancer from inflammatory bowel disease, and hepatocellular carcinoma from hepatitis virus infection [43]. Cancer cells can interact with surrounding basal and inflammatory cells to create an inflammatory TME that promotes tumorigenesis in vivo. At the same time, chronic inflammation in TME blocks anti-tumor immunity, thus providing advantages for tumour development [44].

In addition, reactive oxygen/nitrogen species (ROS/RNS) that are produced from inflammatory cells result in mutagenic DNA lesions leading to cancer genesis [45]. ROS/RNS oxidize guanine into the unstable 8-nitro-guanine and mutagenic 8-oxo-guanine, a production that easily causes nucleotide mispairing [46]. ROS/RNS can also damage lipids, nucleic acids, and proteins through multiple pathways, resulting in repeated tissue damage and repair. Moreover, cancer stem cells are generated from human stem cells via multiple mutations caused by ROS/RNS in TME [47].

Indeed, tumorigenesis stimulates and promotes an inflammatory response, suggesting a mutual reinforcement relationship rather than a unidirectional connection. One of the features of carcinoma is the disruption of intrinsic tumor suppression [48]. Tp53, a frequently mutated tumor suppressor, encodes a crucial activator of inflammation p53 protein. Dysfunctional p53 protein leads to overexpression of inflammatory genes dependent on nuclear factor kappa B (NF-κB) [49]. Besides, the cancer cells can recruit types of immune-inflammatory cells via the expression of cytokines. Studies have shown increased concentrations of multiple cytokines, including interleukin 6, TNFα, interleukin 8, the cytokines MIF, TGFβ, interleukin 10 and interleukin 18 in patients with cancer [50–52].

### Strengths and limitations

Our study has several strengths. To our knowledge, it included the largest number of circulating inflammatory parameters. Meanwhile, we studied the progression (FIGO stage, histologic invasion) as well as long-term outcomes (overall survival and progress-free survival) of endometrial cancer in a long follow-up duration. We conducted a two-sample Mendelian randomization study using 41 inflammatory cytokines and data on endometrial cancer from the GWAS of Finns and 17 other cohorts.

There are limitations in our study. Observational retrospective study design may introduce selection bias. Second, it is a single-center study with participants from one race. The generalization of our findings to the global population should proceed cautiously. Third, confounders such as drinking, smoking, and procreation status were not included in our study.

## Conclusions

Our cohort study indicated that inflammatory level was associated with the progression and long-term outcomes of endometrial cancer. The MR study did not find solid evidence to indicate a causal relationship between inflammatory cytokines and EC. Our study contributes to expanding evidence on the involvement of inflammation in endometrial cancer. In clinical practice, an evaluation system for the inflammation level consisting of various inflammatory indicators should be established. Inflammation level should be considered when predicting tumor grade and prognosis in patients with EC. Finally, targeting inflammation could be a potential therapy for endometrial cancer patients.

### Electronic supplementary material

Below is the link to the electronic supplementary material.

Supplementary Material 1

Supplementary Material 2

Supplementary Material 3

Supplementary Material 4

Supplementary Material 5

## Data Availability

The original data for analysis are presented in the text and supplementary materials. Further reasonable requests for original data supporting the results of our study are available from the corresponding author. The summary statistics GWAS data for inflammatory cytokines and endometrial cancer can be open-assessed from https://gwas.mrcieu.ac.uk/.

## Abbreviations

EC: Endometrial cancer
MR: Mendelian randomization
SNPs: Single-nucleotide polymorphisms
GWAS: Genome-wide association study
FIGO: International Federation of Gynecology and Obstetrics
OR: Odds ratio
WBC: White blood cell
CRP: C-reactive protein
SII: Systemic immune-inflammation
OS: Overall survival
PFS: Progression-free survival
LASSO regression: Least absolute shrinkage and selection operator regression
MLH1: MutL homolog 1
MSH2: MutS homolog 2
MSH6: MutS homolog 6
PMS2: PMS1 homolog 2
BRCA1: BReast CAncer gene 1
BRCA2: BReast CAncer gene 2
RR: Risk ratio
CI: Confidence interval
IGF-1: Insulin growth factor-1
SHBG: Sex hormone binding globulin
DC: Dendritic cell
TME: Tumor microenvironment
NLR: Neutrophil to Lymphocyte Ratio
MPV: Mean platelet volume
IVW: Inverse variance weighted
BMI: Body mass index
STROBE: Strengthening the Reporting of Observational Studies in Epidemiology
LC: Lymphocyte counts
MC: Monocyte counts
PLT: Platelet counts
PDW: Platelet distribution width
PCT: Plateletcrit
PLR: Platelet-to-lymphocyte ratio
IL-10: Interleukin-10
IFN-γ: Interferon-γ
SCGF-β: Stem Cell Growth Factor Beta
MR-PRESSO: MR-pleiotropy residual sum and outlier
ROS/RNS: Reactive oxygen/nitrogen species
DNA: Deoxyribonucleic acid
Tp53: Tumor protein p53
NF-κB: Nuclear factor kappa B
TNFα: Tumor necrosis factor-α
MIF: Migration inhibitory factor
TGFβ: Transforming growth factor beta
