# Supplementary material for: Insights into inflammation and implications for the pathogenesis and long-term outcomes of endometrial cancer: genome-wide surveys and a clinical cohort study
Source: BMC Cancer. 2024 Jul 17;24:846. doi: 10.1186/s12885-024-12630-x (PMC11253470; doi:10.1186/s12885-024-12630-x)
Supplement: Supplementary file 1 — Supplementary Material 1 [file 12885_2024_12630_MOESM1_ESM.docx]

| **Table S1** Sensitivity analysis of Inflammatory cytokines on EC | | | | | | |
| --- | --- | --- | --- | --- | --- | --- |
| **Exposure** | **Outcome** | **Heterogeneity** | | **Horizontal pleiotropy** | | |
|  |  | **Cochran’s Q** | **P** | **Egger intercept** | **P^1^** | **MR-PRESSO^2^** |
| IL−1RA | EC | 1.249 | 0.974 | -0.015 | 0.550 | 0.976 |
| IL−1β | EC | 1.283 | 0.864 | 0.002 | 0.928 | 0.886 |
| IL−2 | EC | 3.688 | 0.931 | -0.011 | 0.419 | 0.886 |
| IL−2RA | EC | 7.045 | 0.217 | -0.036 | 0.103 | 0.326 |
| IL−4 | EC | 5.281 | 0.626 | -0.025 | 0.183 | 0.499 |
| IL−5 | EC | 6.964 | 0.138 | 0.037 | 0.348 | 0.191 |
| IL−6 | EC | 0.512 | 0.972 | -0.011 | 0.625 | 0.963 |
| IL−7 | EC | 10.126 | 0.340 | 0.016 | 0.538 | 0.343 |
| IL−8 | EC | 4.401 | 0.221 | 0.006 | 0.824 | 0.363 |
| IL−9 | EC | 5.915 | 0.315 | -0.023 | 0.556 | 0.352 |
| IL−10 | EC | 10.405 | 0.319 | -0.003 | 0.834 | 0.390 |
| IL−12p70 | EC | 13.205 | 0.154 | -0.035 | 0.012 | 0.248 |
| IL−13 | EC | 2.562 | 0.959 | -0.008 | 0.560 | 0.949 |
| IL−16 | EC | 12.994 | 0.163 | 0.007 | 0.695 | 0.245 |
| IL−17 | EC | 6.872 | 0.650 | 0.014 | 0.485 | 0.679 |
| IL−18 | EC | 13.776 | 0.390 | -0.021 | 0.087 | 0.336 |
| CTACK | EC | 5.722 | 0.678 | -0.018 | 0.327 | 0.697 |
| SDF−1α | EC | 23.677 | 0.003 | -0.021 | 0.433 | 0.053 |
| RANTES | EC | 21.794 | 0.005 | -0.023 | 0.608 | 0.749 |
| MIP1β | EC | 11.454 | 0.832 | -0.001 | 0.890 | 0.837 |
| MIP1α | EC | 1.585 | 0.991 | 0.007 | 0.755 | 0.989 |
| MIG | EC | 16.752 | 0.211 | -0.003 | 0.875 | 0.225 |
| MCP3 | EC | 2.718 | 0.257 | -0.055 | 0.543 | NA |
| MCP1 | EC | 7.853 | 0.853 | 0.011 | 0.391 | 0.871 |
| IP−10 | EC | 3.272 | 0.916 | 0.001 | 0.937 | 0.916 |
| GRPa | EC | 8.282 | 0.406 | 0.023 | 0.251 | 0.508 |
| Eotaxin | EC | 14.721 | 0.472 | 0.001 | 0.969 | 0.532 |
| βNGF | EC | 11.963 | 0.063 | -0.037 | 0.529 | 0.089 |
| VEGF | EC | 6.924 | 0.645 | -0.016 | 0.178 | 0.678 |
| SCGFβ | EC | 8.957 | 0.776 | 0.000 | 0.985 | 0.805 |
| SCF | EC | 17.062 | 0.029 | 0.017 | 0.478 | 0.044 |
| PDGFbb | EC | 7.511 | 0.822 | -0.012 | 0.302 | 0.825 |
| MCSF | EC | 15.237 | 0.033 | 0.047 | 0.131 | 0.041 |
| HGF | EC | 3.337 | 0.765 | 0.000 | 0.985 | 0.771 |
| GCSF | EC | 3.615 | 0.823 | 0.008 | 0.559 | 0.832 |
| bFGF | EC | 2.116 | 0.549 | -0.030 | 0.596 | 0.598 |
| MIF | EC | 0.818 | 0.845 | -0.019 | 0.563 | 0.871 |
| TRAIL | EC | 24.167 | 0.044 | -0.015 | 0.202 | 0.080 |
| TGFα | EC | 4.218 | 0.239 | 0.027 | 0.389 | 0.357 |
| TGFβ | EC | 0.494 | 0.920 | -0.005 | 0.786 | 0.952 |
| IFN -γ | EC | 3.302 | 0.951 | 0.001 | 0.949 | 0.960 |
|  | EC | 1.249 | 0.974 | -0.015 | 0.550 | 0.976 |
