# Supplementary material for: Insights into inflammation and implications for the pathogenesis and long-term outcomes of endometrial cancer: genome-wide surveys and a clinical cohort study
Source: BMC Cancer. 2024 Jul 17;24:846. doi: 10.1186/s12885-024-12630-x (PMC11253470; doi:10.1186/s12885-024-12630-x)
Supplement: Supplementary file 2 — Supplementary Material 2 [file 12885_2024_12630_MOESM2_ESM.docx]

| **Table S2** Univariate regression of subject survival (n = 780) | | | |
| --- | --- | --- | --- |
| **Variable** | **Overall Survival** | | **Risk** |
|  | **HR (95%CI)** | ***P*-value** |  |
| Age at diagnosis | 1.09 (1.06, 1.13) | < 0.001 | high |
| BMI | 1.01 (0.96, 1.07) | 0.777 | high |
| Hypertension | 1.71 (1.12, 2.62) | 0.014 | high |
| Diabetes | 2.07 (1.31, 3.26) | 0.002 | high |
| Age at menarche | 1.16 (1.04, 1.28) | 0.005 | high |
| Menopause | 5.49 (2.53, 11.93) | < 0.001 | high |
| FIGO stage | 6.20 (4.06, 9.49) | < 0.001 | high |
| Histologic invasion | 3.31 (2.16, 5.08) | < 0.001 | high |
| Lymph node metastasis | 4.83 (2.92, 8.11) | < 0.001 | high |
| Postoperative chemotherapy | 3.19 (2.08, 4.88) | < 0.001 | high |
| Surgical procedure | 0.58 (0.36, 0.93) | 0.022 | low |
| NC | 1.10 (1.04, 1.16) | 0.002 | high |
| LC | 0.91 (0.65, 1.27) | 0.573 | low |
| MC | 1.74 (0.84, 3.61) | 0.136 | high |
| PLT | 1.00 (0.99, 1.01) | 0.214 | neutral |
| WBC | 1.13 (1.03, 1.24) | 0.008 | high |
| CRP | 1.03 (1.01, 1.05) | < 0.001 | high |
| PDW | 0.88 (0.79, 0.99) | 0.033 | low |
| MPV | 1.03 (0.88, 1.20) | 0.760 | high |
| PCT | 1.13 (0.93, 1.37) | 0.216 | high |
| PLR | 1.18 (1.00, 1.39) | 0.051 | high |
| NLR | 1.28 (1.11, 1.48) | 0.001 | high |
| SII | 1.25 (1.08, 1.43) | 0.002 | high |
|  | | | |
