# Supplementary material for: Insights into inflammation and implications for the pathogenesis and long-term outcomes of endometrial cancer: genome-wide surveys and a clinical cohort study
Source: BMC Cancer. 2024 Jul 17;24:846. doi: 10.1186/s12885-024-12630-x (PMC11253470; doi:10.1186/s12885-024-12630-x)
Supplement: Supplementary file 3 — Supplementary Material 3 [file 12885_2024_12630_MOESM3_ESM.docx]

| **Table S3.** Univariate regression of subject progression-free survival (n = 780) | | | |
| --- | --- | --- | --- |
| **Variable** | **Overall PFS** | | **Risk** |
|  | **HR (95%CI)** | ***P*-value** |  |
| Age at diagnosis | 1.03 (0.99, 1.06) | 0.173 | high |
| BMI | 0.96 (0.89, 1.04) | 0.298 | low |
| Hypertension | 1.23 (0.68, 2.22) | 0.499 | high |
| Diabetes | 2.11 (1.12, 3.98) | 0.021 | high |
| Age at menarche | 1.00 (0.86, 1.17) | 0.949 | neutral |
| Menopause | 2.62 (1.16, 5.90) | 0.020 | high |
| FIGO stage | 7.35 (4.04, 13.369) | < 0.001 | high |
| Histologic invasion | 2.11 (1.13, 3.94) | 0.019 | high |
| Lymph node metastasis | 7.44 (3.89, 14.25) | < 0.001 | high |
| Postoperative chemotherapy | 6.98 (3.65, 13.36) | < 0.001 | high |
| Surgical procedure | 0.44 (0.22, 0.88) | 0.020 | low |
| NC | 1.06 (0.96, 1.18) | 0.244 | high |
| LC | 1.40 (0.93, 2.11) | 0.109 | high |
| MC | 1.58 (0.53, 4.71) | 0.407 | high |
| PLT | 1.00 (0.99, 1.01) | 0.482 | neutral |
| WBC | 1.15 (1.01, 1.30) | 0.037 | high |
| CRP | 1.02 (0.99, 1.05) | 0.191 | high |
| PDW | 1.00 (0.84, 1.21) | 0.947 | neutral |
| MPV | 0.96 (0.77, 1.19) | 0.690 | low |
| PCT | 1.05 (0.79, 1.40) | 0.733 | high |
| PLR | 0.97 (0.71, 1.33) | 0.854 | high |
| NLR | 1.09 (0.82, 1.45) | 0.565 | high |
| SII | 1.10 (0.85, 1.44) | 0.463 | high |
|  | | | |
