# Supplementary material for: Insights into inflammation and implications for the pathogenesis and long-term outcomes of endometrial cancer: genome-wide surveys and a clinical cohort study
Source: BMC Cancer. 2024 Jul 17;24:846. doi: 10.1186/s12885-024-12630-x (PMC11253470; doi:10.1186/s12885-024-12630-x)
Supplement: Supplementary file 4 — Supplementary Material 4 [file 12885_2024_12630_MOESM4_ESM.docx]

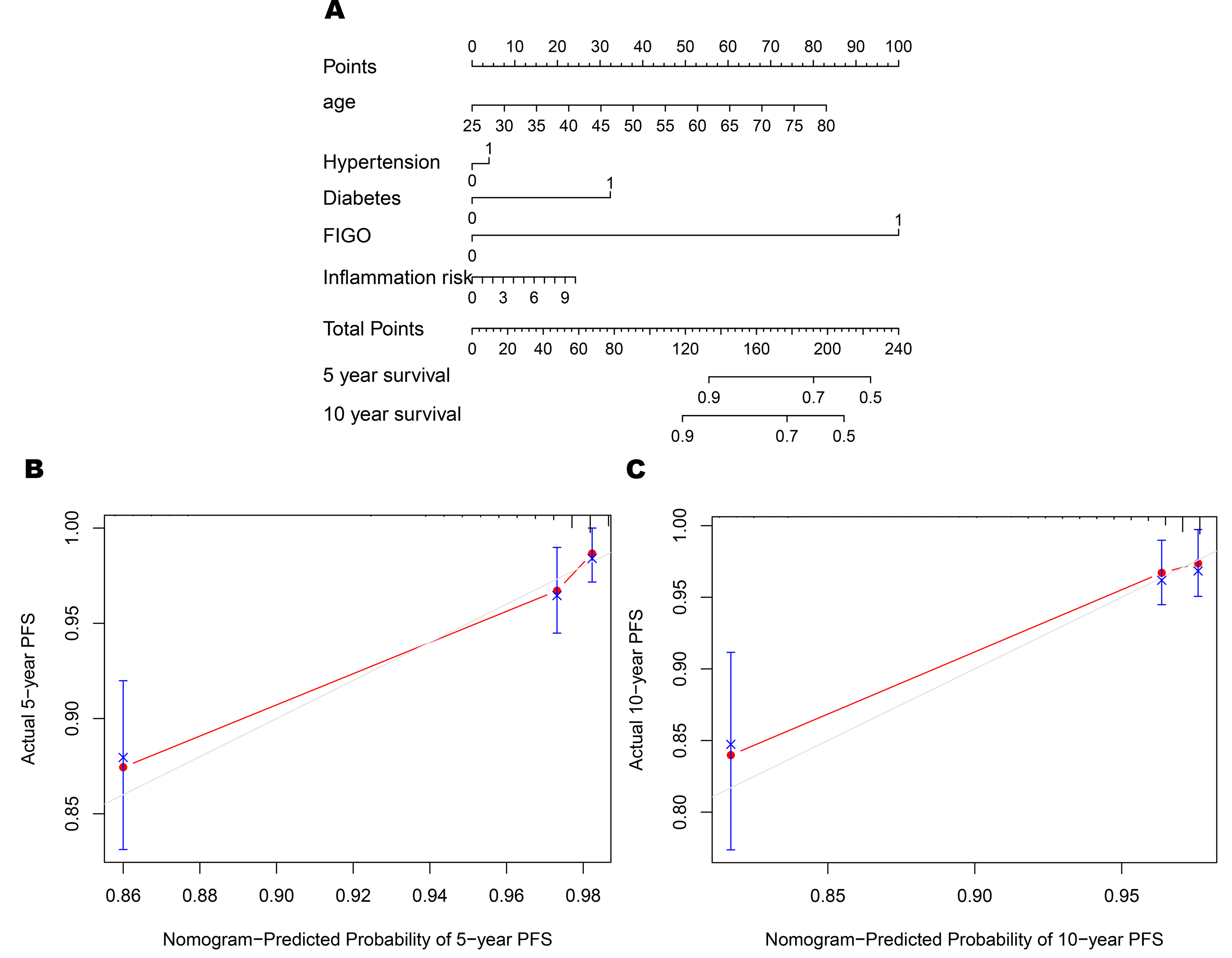


**Table S4.** Nomogram for estimating 5 or 10 years PFS probility for patients with endometrial cancer (A), Calibration curves of the nomograms of 5 (B) or 10 year (C) PFS prediction
